# Association between dietary magnesium intake and gallstones: the mediating role of atherogenic index of plasma

**DOI:** 10.1186/s12944-024-02074-4

**Published:** 2024-03-20

**Authors:** Wenyi Du, Chen Yan, Yinkang Wang, Chen Song, Yunfan Li, Zhiqiang Tian, Yuan Liu, Wei Shen

**Affiliations:** 1 Department of General Surgery, The Affiliated Wuxi People’s Hospital of Nanjing Medical University, Wuxi, China; 2 Wuxi Medical Center of Nanjing Medical University, Wuxi, China; 3 Medical Imaging Centre, Tengzhou Central People’s Hospital, Jining Medical College, Shandong, China; 4 Department of General Surgery, Affiliated Hospital of Jiangsu University, Zhenjiang, China

**Keywords:** Gallstones, Dietary magnesium, Atherogenic index of plasma, National health and nutrition examination survey

## Abstract

**Background:**

Dyslipidemia and abnormalities in cholesterol metabolism are commonly observed in individuals with gallstone disease. Previous research has demonstrated that dietary magnesium can influence lipid metabolism. The atherogenic index of plasma (AIP) has emerged as a novel lipid marker. This study aimed to examine the possible correlation between dietary magnesium intake and gallstones and the potential mediating role of AIP in US adults.

**Methods:**

A total of 4,841 adults were included in this study from the National Health and Nutrition Examination Survey (NHANES) conducted from 2017 to 2020. A variety of statistical techniques such as logistic regression, subgroup analysis, smoothed curve fitting, and causal mediation analysis were utilized to analyze the information collected from the participants.

**Results:**

In the fully adjusted model, a statistically noteworthy inverse relationship was observed between dietary magnesium intake and the presence of gallstones, as indicated by an odds ratio (OR) of 0.58 and a 95% confidence interval (CI) of (0.42, 0.81). Causal intermediary analysis revealed that the association between magnesium intake and gallstones was partially mediated by AIP, with a mediation ratio of 3.2%.

**Conclusion:**

According to this study, dietary magnesium intake had a significant linear negative association with the prevalence of gallstones, in which AIP played a mediating role. This discovery offers novel perspectives on the prevention and management of gallstones.

## Introduction

Gallstones represent a prevalent gastrointestinal ailment impacting 10-15% of the global populace, with varying incidence rates among nations [1]. In the United States, approximately 10-20% of adults currently harbor gallstones, a figure that is on the rise [2]. The annual cost of preventing and treating gallstone disease in the United States has been calculated to be approximately $62 billion, placing a significant financial burden on the healthcare economy [3]. Complications related to gallstones, including cholecystitis, acute suppurative cholangitis, and pancreatitis, manifest in approximately 20% to 40% of individuals with gallstones, with an annual occurrence rate ranging from 1% to 3% [1, 4]. In addition, gallstones increase the likelihood of gallbladder cancer and the prognosis for gallbladder cancer is poor [5, 6]. Therefore, valid and controlled clinical indicators are essential to predict or prevent the development of gallstones.

The atherogenic index of plasma (AIP) was first proposed by Dobiásová M and colleagues in 2001 and was initially used to monitor dyslipidemia and to assess the degree of atherosclerosis [7]. This index combines levels of triglycerides (TG) and high-density lipoprotein cholesterol (HDL-C), reflecting not only the ratio of pro-atherosclerotic to protective lipids in plasma but also the particle size and rate of esterification of HDL-C particle size and rate of esterification [8]. Evidence suggests that AIP is more specific than a single lipid marker in the diagnosis and detection of cardiovascular and dyslipidemia diseases [8, 9]. Consequently, AIP has emerged as a critical parameter within the field of lipidology, subject to ongoing research aimed at uncovering its broader clinical applications.

Magnesium, represented by the chemical symbol Mg, is naturally present in a regular diet and ranks as the body's fourth most plentiful mineral [10]. The decrease in dietary magnesium consumption in developed countries in recent years can be linked to the increasing occurrence of diets low in magnesium, which include processed and fast foods. As a result, a considerable portion of the American population does not meet the recommended average amount of magnesium intake [11]. Inadequate dietary magnesium intake has been linked to the development of multi-system diseases, including cognitive impairment, type 2 diabetes, depression, and metabolic syndrome [12–14]. Additionally, magnesium modulates systemic inflammation, modifies dyslipidemia, and mitigates the likelihood of hypertension and cardiovascular disease [15]. Nevertheless, few studies have reported a correlation between the consumption of magnesium-rich foods and the occurrence of gallstones. The precise mechanism responsible for this correlation remains uncertain, as magnesium influences lipid levels.

Thus, the novelty of this study resides in its unique investigation of data from a public database to validate the relationship between dietary magnesium and gallstones, as well as the moderating influence of AIP, within a substantial sample population. The primary objective was to determine if there exists a disparity in magnesium consumption between individuals with and without gallstones to propose fresh recommendations for clinical gallstone treatment. Additionally, the secondary goal was to analyze whether dietary magnesium mediated the intended association through the lipid parameter AIP.

## Methodology of the study

### Study design and subject inclusion and exclusion

The survey data utilized in this study is derived from the U.S. National Health and Nutrition Examination Survey (NHANES) conducted biennially between 2017 and 2020. NHANES offers a comprehensive evaluation and analysis of the overall nutrition and health condition of the American populace, overseen by the National Center for Health Statistics (NCHS). The NHANES employs a dynamic, multi-stage, and probability-based complex sampling design through household interviews at respondents' residences, physical examinations at mobile health screening centers, and specialized tests in laboratories, thus ensuring the accuracy and representativeness of the data [16, 17]. Approval for the survey was obtained from the Ethics Committee of the Centers for Disease Control and Prevention (CDC), with all participants providing informed consent. Comprehensive information regarding the dataset, documentation, and protocols can be accessed at no cost on the NHANES website [18].

**Fig. 1:**
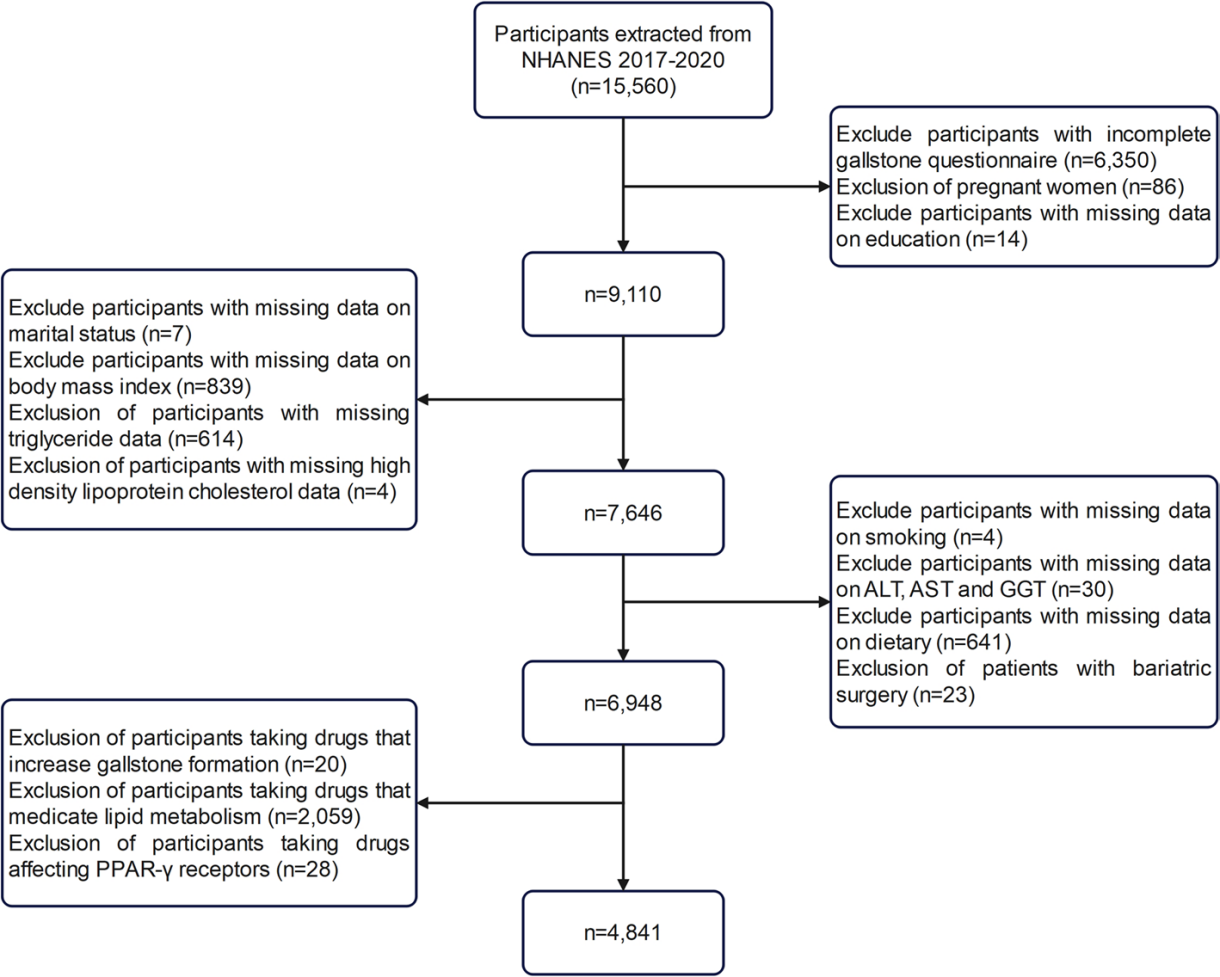
Flowchart of participant selection from NHANES 2017-2020

Initially, this study excluded individuals with incomplete gallstone information on the Medical Health Questionnaire (*n* = 6,350). Subsequently, participants with missing data on lipid markers, liver enzymes, and dietary information were excluded. Furthermore, 23 patients who had previously undergone bariatric surgery were eliminated from the analysis. After taking into account pharmacological considerations, individuals using medications that elevate the likelihood of gallstones and impact lipid metabolism (such as glucagon-like peptide-1 receptor agonists, lipid-lowering drugs, and peroxisome proliferator-activated receptor-gamma-affecting drugs) were excluded from the study. Ultimately, a total of 4,841 subjects were selected for inclusion in this analysis. The detailed procedure of subject screening is illustrated in Figure 1, offering a comprehensive account of the process.

### Definition of gallstones

In a survey conducted by trained professional interviewers, respondents were asked the question: "Has a doctor or other health professional ever diagnosed you with gallstones?" The findings of the survey revealed that individuals who responded affirmatively were classified as having gallstones, while those who responded negatively were deemed to be free of gallstone disease. This simple and easy method has been employed in prior research studies [19, 20].

### Definition of AIP

HDL-C and TG data were assayed by the University of Minnesota laboratory under strict quality control and supervision. AIP was calculated by applying the formula: AIP = Log [TG (mmol/L) / HDL-C (mmol/L)] [21]. In the present study, AIP was recognized as a mediating variable that held particular significance.

### Dietary magnesium intake

A multichannel approach was used to collect data on magnesium intake from 24-hour dietary recalls. The respondent-driven approach gathers precise data on every food and drink consumed by an individual within 24 hours, from midnight to midnight. Each participant was asked to participate in two 24-hour total nutritional intake recall interviews. The initial recall involved an in-person interview with an investigator at a mobile screening facility, while the subsequent recall took place via telephone within a span of 3 to 10 days [22]. Considering that some participants regularly consumed magnesium-containing supplements, their magnesium intake from dietary supplements was added to their total magnesium intake. In cases where an individual completed two 24-hour dietary records, the average magnesium intake from both records was utilized. Otherwise, the data from the initial 24-hour dietary record was employed.

### Ascertainment of covariates

To thoroughly examine the possible link between magnesium consumption and gallstones, this study took into account a wide range of covariates such as demographic information, diet details, physical measurements, laboratory tests, and questionnaire responses. NHANES gathered 24-hour dietary data from participants between 2017 and March 2020 with the help of trained interviewers. Dietary intake was obtained from two 24-hour dietary records and averaged twice [23, 24]. The physical examination module incorporated measurements of subjects' blood pressure and body mass index (BMI), in addition to other relevant parameters. Laboratory tests were conducted on lipid and biochemical parameters obtained from participants during the survey period. Liver enzymes, specifically alanine aminotransferase (ALT), aspartate aminotransferase (AST), and glutamyl transferase (GGT), were included as covariates to provide additional insight into the liver health of the subjects. Hypertension was diagnosed when the subject had a systolic blood pressure ≥140 mmHg and a diastolic blood pressure ≥90 mmHg at the mean of three measurements of body circulatory arterial pressure taken at rest or was told or self-reported by a healthcare professional to be hypertensive and/or was taking antihypertensive medication [25]. Diabetes mellitus meets the following criteria: fasting blood glucose level ≥ 7.0 mmol/L, or glycosylated hemoglobin≥ 6.5%, has been informed by a doctor or admitted to having diabetes mellitus, and/or is using medication or insulin to control blood glucose [26]. The diagnosis of fatty liver is determined through the use of vibration-controlled transient elastography, a non-invasive and painless procedure that measures controlled attenuation parameters (CAP). CAP ≥274 dB/m indicates a fatty liver state and CAP ≥302 dB/m is defined as severe fatty liver [27, 28]. Based on the self-reported questionnaire, smoking behaviors were classified into three categories: never-smokers, former smokers, and current smokers. Based on previous studies, alcohol consumption was categorized into four levels: heavy drinking (≥4 drinks/day for men, ≥3 drinks/day for women, or ≥5 days of drinking in a month), moderate drinking (≥3 drinks/day for men, ≥2 drinks/day for women, or ≥2 days of drinking in a month), mild drinking (≤2 drinks/day for men, ≤1 drink/day for women, and ≥12 drinks in a year), and never-drinking (total number of drinks in a year <12, and dietary alcohol content of 0%) [29].

### Statistical analysis

Continuous variables collected from participants were tested for normality according to the characteristics of the data, and data obeying a normal distribution were expressed as mean ± standard deviation (SD), while data not obeying a normal distribution were expressed as median and interquartile range (IQR). Qualitative variables were expressed as relative numbers or percentages. To avoid multicollinearity, variance inflation factor (VIF) and tolerance were used to assess the covariates, and the VIF of all covariates included in this study was less than 5, indicating the absence of severe multicollinearity [30]. Due to the skewed distribution of dietary magnesium intake, data were transformed using natural logarithms before analyses and analyzed as continuous variables (per 1-SD increment) in multivariate models and mediation analyses. Differences between subjects grouped by quartiles of magnesium intake were compared in multivariable logistic regression, using quartile 1 (Q1) as the reference group. Four logistic models were developed for the analyses: Model 1 was not adjusted for any confounding variables and was a Univariate analysis. Model 2 was adjusted for the main demographic variables (sex, age, and ethnicity). Model 3 adds smoking, alcohol consumption, marital status, education, ratio of household income to poverty (PIR), BMI, hypertension, fatty liver, diabetes, lipids, and liver enzymes to Model 2. Considering the confounding effect of dietary factors on gallstones, Model 4 further incorporated micronutrients such as carbohydrates, proteins, fats, water, calcium, iron, and zinc [31]. In addition, subgroup analyses were conducted to examine the presence of heterogeneity and interactions in specific groups of the population. To visualize more closely the linear or non-linear correlation between gallstones and magnesium intake, curves were fitted using restricted cubic spline (RCS). Finally, mediation analyses were performed using the mediation package, and confidence intervals for the mediating effect were assessed using the Bootstrap method to determine the proportion of the mediating effect accounted for by AIP. Using these statistical methods, the possible causal relationship between magnesium intake and gallstones can be examined more broadly. All statistical analyses were based on R software (version 4.22) and EmpowerStats (version 4.0). Graphs were plotted using the ggplot2 package and image beautification was done using the ggprism package. Statistical significance was defined as a *P* value below 0.05.

## Results

### Clinical baseline features of the subjects

According to their gallstone status, Table 1 summarizes the clinical baseline features of the subjects. The analysis revealed a statistically significant disparity in gender distribution, with a greater representation of females in the gallstone group (*P* < 0.001). Furthermore, the gallstone group exhibited a slightly higher median age (52 years versus 43 years, *P* < 0.001). Significant disparities were noted in biochemical and dietary parameters, including triglyceride levels and magnesium intake (*p* < 0.001), indicating a potential correlation between metabolism, diet, and the development of gallstones. Moreover, the *P*-values for the levels of alanine aminotransferase, glutamine aminotransferase, and glutamyl transpeptidase were not significant, suggesting that liver function was comparable between the two groups of subjects.

**Table 1:** Baseline characteristics of the gallstones group versus the no-gallstones group.

**Characteristics**	**Overall, *N* = 4,841**	**Gallstones, *N* = 413**	**No gallstones, *N*= 4,428**	***P* value**
Gender, n (%)				<0.001
Male	2,277 (47.04%)	101 (24.46%)	2,176 (49.14%)	
Female	2,564 (52.96%)	312 (75.54%)	2,252 (50.86%)	
Age	44 (32, 58)	52 (39, 64)	43 (31, 57)	<0.001
Race, n (%)				<0.001
Mexican American	632 (13.06%)	59 (14.29%)	573 (12.94%)	
Non-Hispanic White	1,664 (34.37%)	174 (42.13%)	1,490 (33.65%)	
Non-Hispanic Black	1,255 (25.92%)	81 (19.61%)	1,174 (26.51%)	
Other Races	1,290 (26.65%)	99 (23.97%)	1,191 (26.90%)	
Education level, n (%)				0.823
Grades 0–12	802 (16.57%)	72 (17.43%)	730 (16.49%)	
High school graduate/GED	1,172 (24.21%)	102 (24.70%)	1,070 (24.16%)	
Some college or above	2,867 (59.22%)	239 (57.87%)	2,628 (59.35%)	
Marital status, n (%)				<0.001
Married/Living with Partner	2,788 (57.59%)	254 (61.50%)	2,534 (57.23%)	
Widowed/Divorced/Separated	903 (18.65%)	95 (23.00%)	808 (18.25%)	
Unmarried	1,150 (23.76%)	64 (15.50%)	1,086 (24.53%)	
PIR, n (%)				0.495
<2	1,944 (40.16%)	161 (38.98%)	1,783 (40.27%)	
≥2	2,305 (47.61%)	207 (50.12%)	2,098 (47.38%)	
Unclear	592 (12.23%)	45 (10.90%)	547 (12.35%)	
Hypertension, n (%)				<0.001
Yes	1,625 (33.57%)	199 (48.18%)	1,426 (32.20%)	
No	3,216 (66.43%)	214 (51.82%)	3,002 (67.80%)	
Diabetes, n (%)				<0.001
Yes	468 (9.67%)	72 (17.43%)	396 (8.94%)	
No	4,373 (90.33%)	341 (82.57%)	4,032 (91.06%)	
Fatty liver, n (%)				<0.001
No	2,962 (61.19%)	200 (48.43%)	2,762 (62.38%)	
Mild	699 (14.44%)	74 (17.92%)	625 (14.11%)	
Severe	1,180 (24.38%)	139 (33.66%)	1,041 (23.51%)	
Smoking, n (%)				0.062
Current smokers	952 (19.67%)	77 (18.64%)	875 (19.76%)	
Former smokers	958 (19.79%)	100 (24.21%)	858 (19.38%)	
Never smokers	2,931 (60.55%)	236 (57.14%)	2,695 (60.86%)	
Alcohol consumption, n (%)				0.003
Heavy	1,657 (34.23%)	109 (26.39%)	1,548 (34.96%)	
Moderate	483 (9.98%)	44 (10.65%)	439 (9.91%)	
Mild	843 (17.41%)	72 (17.43%)	771 (17.41%)	
Never	1,858 (38.38%)	188 (45.52%)	1,670 (37.71%)	
BMI (kg/m^2^)	28 (24, 33)	32 (27, 38)	28 (24, 33)	<0.001
TG (mmol/L)	1.19 (0.84, 1.73)	1.34 (0.95, 1.82)	1.16 (0.84, 1.72)	<0.001
HDL-C (mmol/L)	1.32 (1.11, 1.60)	1.32 (1.11, 1.58)	1.32 (1.09, 1.63)	0.626
TC (mmol/L)	4.76 (4.16, 5.43)	4.76 (4.19, 5.43)	4.76 (4.16, 5.43)	0.689
ALT (U/L)	17 (13, 25)	17 (13, 25)	17 (13, 25)	0.837
AST (U/L)	19 (16, 24)	19 (15, 23)	19 (16, 24)	0.064
GGT(IU/L)	20 (14, 31)	20 (15, 33)	20 (14, 31)	0.255
Protein intake (g)	73 (55, 97)	64 (49, 85)	74 (55, 98)	<0.001
Carbohydrate intake (g)	225 (167, 301)	214 (154, 279)	226 (168, 303)	0.002
Fat intake (g)	77 (55, 107)	71 (53, 99)	78 (55, 107)	0.006
Water intake (g)	2,558 (1,889, 3,408)	2,415 (1,790, 3,313)	2,567 (1,896, 3,414)	0.046
Magnesium intake(mg)	275 (201, 376)	260 (184, 345)	277 (202, 379)	<0.001
Calcium intake(mg)	799 (554, 1,116)	793 (536, 1,052)	801 (555, 1,121)	0.205
Iron intake(mg)	12 (9, 16)	11 (8, 15)	12 (9, 17)	<0.001
Zinc intake(mg)	9.2 (6.7, 12.7)	8.2 (6.1, 11.4)	9.3 (6.7, 12.8)	<0.001
AIP	-0.06 (-0.26, 0.16)	-0.01 (-0.19, 0.20)	-0.06 (-0.27, 0.16)	<0.001

Median (IQR) for continuous variables; N (%) for categorical variables

*Abbreviations*: *PIR* Ratio of household income to poverty, *GED* General equivalency diploma, *BMI* Body mass index, *TG* Triglyceride, *HDL-C* High density lipoprotein cholesterol, *TC* Total cholesterol, *ALT* Alanine Aminotransferase, *AST* Aspartate Aminotransferase, *GGT* Gamma Glutamyl Transferase; *AIP* Atherogenic index of plasma

Table 2 demonstrates the classification of subjects into quartiles based on dietary magnesium intake. It can be seen from the table that participants in the fourth quartile (6.44%) had a significantly lower incidence of gallstones compared to the first quartile (10.50%).

**Table 2:** Baseline characteristics of the study population based on dietary magnesium intake

Characteristics	Magnesium intake (mg)				*P* value
	Q1 [9.5,201), *N* = 1,210	Q2 [201,275), *N* = 1,210	Q3 [275,376), *N* = 1,209	Q4 [376,2273], *N* = 1,212	
Gender, n (%)					<0.001
Male	423 (34.96%)	493 (40.74%)	606 (50.12%)	755 (62.29%)	
Female	787 (65.04%)	717 (59.26%)	603 (49.88%)	457 (37.71%)	
Age	42 (29, 59)	42 (31, 56)	44 (32, 59)	46 (33, 59)	<0.001
Race, n (%)					<0.001
Mexican American	103 (8.51%)	141 (11.65%)	184 (15.22%)	204 (16.83%)	
Non-Hispanic White	352 (29.09%)	414 (34.21%)	431 (35.65%)	467 (38.53%)	
Non-Hispanic Black	463 (38.26%)	324 (26.78%)	274 (22.66%)	194 (16.01%)	
Other Races	292 (24.13%)	331 (27.36%)	320 (26.47%)	347 (28.63%)	
Education level, n (%)					<0.001
Grades 0–12	242 (20.00%)	186 (15.37%)	173 (14.31%)	201 (16.58%)	
High school graduate/GED	379 (31.32%)	280 (23.14%)	295 (24.40%)	218 (17.99%)	
Some college or above	589 (48.68%)	744 (61.49%)	741 (61.29%)	793 (65.43%)	
Marital status, n (%)					<0.001
Married/Living with Partner	593 (49.01%)	695 (57.44%)	743 (61.46%)	757 (62.46%)	
Widowed/Divorced/Separated	247 (20.41%)	222 (18.35%)	208 (17.20%)	226 (18.65%)	
Unmarried	370 (30.58%)	293 (24.21%)	258 (21.34%)	229 (18.89%)	
PIR, n (%)					<0.001
<2	623 (51.49%)	474 (39.17%)	444 (36.72%)	403 (33.25%)	
≥2	450 (37.19%)	583 (48.18%)	612 (50.62%)	660 (54.46%)	
Unclear	137 (11.32%)	153 (12.64%)	153 (12.66%)	149 (12.29%)	
Hypertension, n (%)					0.362
Yes	429 (35.45%)	388 (32.07%)	403 (33.33%)	405 (33.42%)	
No	781 (64.55%)	822 (67.93%)	806 (66.67%)	807 (66.58%)	
Diabetes, n (%)					0.547
Yes	127 (10.50%)	121 (10.00%)	112 (9.26%)	108 (8.91%)	
No	1,083 (89.50%)	1,089 (90.00%)	1,097 (90.74%)	1,104 (91.09%)	
Fatty liver, n (%)					0.102
No	774 (63.97%)	723 (59.75%)	725 (59.97%)	740 (61.06%)	
Mild	167 (13.80%)	162 (13.39%)	183 (15.14%)	187 (15.43%)	
Severe	269 (22.23%)	325 (26.86%)	301 (24.90%)	285 (23.51%)	
Gallstones					0.005
Yes	127 (10.50%)	105 (8.68%)	103 (8.52%)	78 (6.44%)	
No	1,083 (89.50%)	1,105 (91.32%)	1,106 (91.48%)	1,134 (93.56%)	
Smoking, n (%)					<0.001
Current smokers	301 (24.88%)	235 (19.42%)	206 (17.04%)	210 (17.33%)	
Former smokers	195 (16.12%)	204 (16.86%)	268 (22.17%)	291 (24.01%)	
Never smokers	714 (59.01%)	771 (63.72%)	735 (60.79%)	711 (58.66%)	
Alcohol consumption, n (%)					<0.001
Heavy	333 (27.52%)	397 (32.81%)	416 (34.41%)	511 (42.16%)	
Moderate	118 (9.75%)	124 (10.25%)	140 (11.58%)	101 (8.33%)	
Mild	209 (17.27%)	226 (18.68%)	216 (17.87%)	192 (15.84%)	
Never	550 (45.45%)	463 (38.26%)	437 (36.15%)	408 (33.66%)	
BMI (kg/m^2^)	29 (24, 35)	29 (25, 34)	28 (24, 33)	28 (24, 32)	<0.001
TG (mmol/L)	1.12 (0.82, 1.60)	1.17 (0.84, 1.69)	1.22 (0.86, 1.82)	1.22 (0.85, 1.83)	<0.001
HDL-C (mmol/L)	1.34 (1.11, 1.58)	1.34 (1.10, 1.63)	1.32 (1.11, 1.60)	1.34 (1.11, 1.63)	0.545
TC (mmol/L)	4.68 (4.09, 5.38)	4.76 (4.16, 5.38)	4.84 (4.22, 5.48)	4.76 (4.16, 5.46)	0.005
ALT (U/L)	15 (11, 22)	17 (12, 25)	17 (13, 26)	20 (14, 28)	<0.001
AST (U/L)	18 (15, 22)	18 (15, 23)	19 (16, 24)	21 (17, 25)	<0.001
GGT(IU/L)	19 (14, 30)	20 (14, 31)	21 (14, 31)	21 (14, 32)	0.102
Protein intake (g)	50 (38, 63)	69 (55, 83)	84 (67, 103)	102 (79, 130)	<0.001
Carbohydrate intake (g)	159 (115, 203)	208 (167, 261)	252 (199, 315)	303 (234, 384)	<0.001
Fat intake (g)	55 (38, 71)	72 (55, 94)	88 (66, 115)	103 (77, 137)	<0.001
Water intake (g)	1,784 (1,332, 2,319)	2,361 (1,843, 3,034)	2,751 (2,220, 3,484)	3,416 (2,704, 4,398)	<0.001
Calcium intake(mg)	501 (342, 695)	741 (544, 946)	915 (709, 1,201)	1,161 (847, 1,562)	<0.001
Iron intake(mg)	8 (6, 10)	11 (9, 14)	14 (11, 18)	17 (13, 22)	<0.001
Zinc intake(mg)	6.0 (4.3, 7.8)	8.4 (6.6, 10.6)	10.4 (8.1, 13.5)	13.3 (10.2, 17.8)	<0.001
AIP	-0.08 (-0.26, 0.12)	-0.06 (-0.28, 0.15)	-0.04 (-0.24, 0.19)	-0.05 (-0.26, 0.20)	0.013

Median (IQR) for continuous variables; N (%) for categorical variables

*Abbreviations*: *PIR* Ratio of household income to poverty; *GED* General equivalency diploma, *BMI* Body mass index, *TG* Triglyceride, *HDL-C* High density lipoprotein cholesterol, *TC* Total cholesterol, *ALT* Alanine Aminotransferase, *AST* Aspartate Aminotransferase, *GGT* Gamma Glutamyl Transferase, *AIP* Atherogenic index of plasma

### Logistic regression analysis

The results of the logistic regression analyses for the four models are summarized in Table 3. Model 1, unadjusted for covariates, revealed a negative correlation between magnesium intake and gallstones, with an odds ratio of 0.66 (95% CI: 0.54-0.81; *P* < 0.0001). The odds ratio of Model 2, after controlling for the primary demographic covariates, closely aligns with those of Model 1. Model 3 expanded upon Model 2 by including additional variables such as education level, BMI, smoking habits, marital status, PIR, alcohol consumption, underlying diseases (hypertension, diabetes, fatty liver), and biochemical indicators (TG, HDL-C, total cholesterol, ALT, AST, and GGT). Despite these adjustments, the inverse relationship between magnesium intake and gallstones remained statistically significant (OR = 0.73; 95% CI: 0.58-0.91; *P* = 0.0048). Interestingly, this negative correlation became even more pronounced in Model 4 after controlling for all confounders (OR = 0.58; *P* = 0.0015). This indicates that each unit increase in dietary magnesium intake (converted to natural logarithms) was associated with a 42% reduction in the incidence of gallstones. Further dividing magnesium intake into quartiles, the negative association between magnesium and gallstones remained. In model 4, the incidence of gallstones was 40% lower in the population in the highest dietary magnesium intake group (Q4) compared with the lowest intake group (Q1).

**Table 3:** Odds ratios and 95% confidence intervals for gallstones according to dietary magnesium intake

	**OR (95%CI), *P* value**			
**Exposure**	**Model 1**	**Model 2**	**Model 3**	**Model 4**
**Per 1-SD increment **^**a**^	0.66 (0.54, 0.81) <0.0001	0.68 (0.55, 0.85) 0.0004	0.73 (0.58, 0.91) 0.0048	0.58 (0.42, 0.81) 0.0015
**Magnesium intake (Quartile)**				
**Q1 [9.5,201)**	Reference	Reference	Reference	Reference
**Q2 [201,275)**	0.81 (0.62, 1.06) 0.1293	0.82 (0.62, 1.09) 0.1706	0.83 (0.62, 1.11) 0.2141	0.80 (0.58, 1.09) 0.1534
**Q3 [275,376)**	0.79 (0.60, 1.04) 0.0981	0.81 (0.61, 1.07) 0.1406	0.87 (0.65, 1.17) 0.3482	0.80 (0.56, 1.15) 0.2265
**Q4 [376,2273]**	0.59 (0.44, 0.79) 0.0004	0.64 (0.47, 0.87) 0.0045	0.69 (0.50, 0.96) 0.0259	0.60 (0.39, 0.94) 0.0243
***P* for trend**	0.63 (0.49, 0.82) 0.0005	0.68 (0.52, 0.89) 0.0055	0.74 (0.55, 0.98) 0.0383	0.65 (0.44, 0.97) 0.0341

^a^ Magnesium intake was log-transformed before analysis

OR: odds ratio; 95% Cl: 95% confidence interval

Model 1: no adjustment for covariates

Model 2: adjusted for sex, age, and race

Model 3: adjusted for sex, age, race, education level, marital status, PIR, BMI, hypertension, diabetes mellitus, fatty liver, smoking, alcohol consumption, TG, HDL-C, TC, ALT, AST, and GGT

Model 4: adjusted for sex, age, race, education level, marital status, PIR, BMI, hypertension, diabetes mellitus, fatty liver, smoking, alcohol consumption, TG, HDL-C, TC, ALT, AST, GGT, protein intake, carbohydrate intake, fat intake, water intake, calcium intake, iron intake, and zinc intake

AIP and gallstones are correlated according to multivariate logistic regression provided in Table 4. According to the initial model, AIP levels were significantly and positively associated with gallstones (OR = 1.61; 95% CI: 1.17-2.20; *P* = 0.0032). Notably, the stable positive correlation in Model 2 was particularly evident when sex, age, and race were considered (OR = 2.27; *P* < 0.0001). Model 3 remained stable after continuing to include the variables of smoking, alcohol consumption, education level, marital status, PIR, total blood cholesterol, hypertension, fatty liver, diabetes, and liver enzymes. Subsequently, Model 4 incorporated additional dietary variables such as protein, carbohydrate, fat, water, calcium, iron, and zinc, and the notable positive association between AIP levels and gallstone prevalence persisted (OR = 1.57; 95% CI: 1.05-2.35; *P* = 0.0283).

**Table 4:** Multiple logistic regression of the association between AIP and gallstones

	**OR (95%CI), *P* value**			
**Exposure**	**Model 1**	**Model 2**	**Model 3**	**Model 4**
**AIP (Continuous)**	1.61 (1.17, 2.20) 0.0032	2.27 (1.59, 3.23) <0.0001	1.55 (1.04, 2.31) 0.0318	1.57 (1.05, 2.35) 0.0283
**AIP (Quartile)**				
**Q1 [-0.959, -0.259)**	Reference	Reference	Reference	Reference
**Q2 [-0.259, -0.0588)**	1.39 (1.01, 1.91) 0.0404	1.38 (1.00, 1.90) 0.0509	1.25 (0.90, 1.73) 0.1875	1.25 (0.90, 1.74) 0.1800
**Q3 [-0.0588,0.165)**	1.82 (1.35, 2.46) <0.0001	1.90 (1.39, 2.59) <0.0001	1.52 (1.09, 2.11) 0.0124	1.51 (1.08, 2.09) 0.0149
**Q4 [0.165,1.39]**	1.70 (1.26, 2.31) 0.0006	2.08 (1.50, 2.86) <0.0001	1.54 (1.09, 2.19) 0.0153	1.57 (1.10, 2.23) 0.0127
***P* for trend**	2.03 (1.39, 2.98) 0.0003	2.76 (1.82, 4.17) <0.0001	1.81 (1.14, 2.86) 0.0118	1.84 (1.16, 2.92) 0.0100

AIP was converted from a continuous variable to a categorical variable (quartiles) in multiple logistic regression analyses.

OR: odds ratio; 95% Cl: 95% confidence interval

Model 1: no adjustment for covariates

Model 2: adjusted for sex, age, and race

Model 3: adjusted for sex, age, race, education level, marital status, PIR, hypertension, diabetes mellitus, fatty liver, smoking, alcohol consumption, TC, ALT, AST, and GGT

Model 4: adjusted for sex, age, race, education level, marital status, PIR, hypertension, diabetes mellitus, fatty liver, smoking, alcohol consumption, TC, ALT, AST, GGT, protein intake, carbohydrate intake, fat intake, water intake, calcium intake, iron intake, and zinc intake

### Subgroup analysis

**Fig. 2:**
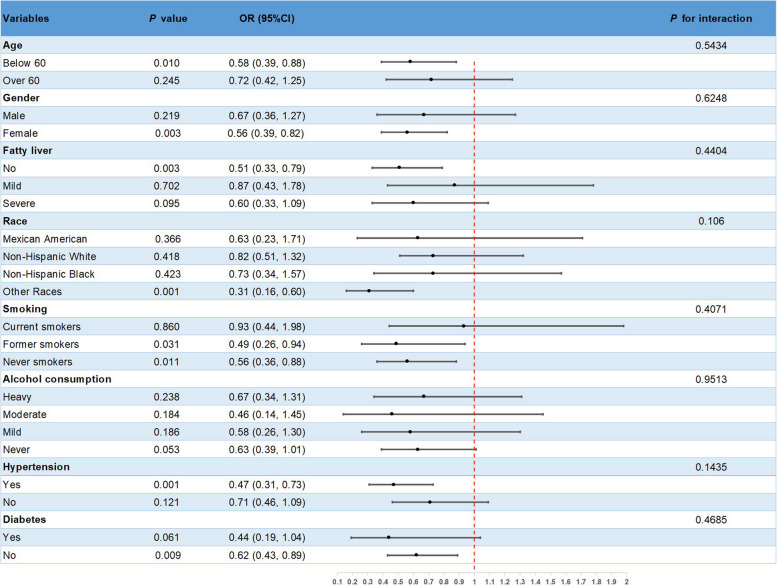
Subgroup analysis for the association between dietary magnesium intake and gallstones

A subgroup analysis was conducted on Model 4 to investigate potential variations in the relationship between gallstones and dietary magnesium intake among different populations. The findings are presented in Figure 2. It is noteworthy that the inverse correlation between magnesium intake and the incidence of gallstones was notably significant in subgroups consisting of females, individuals under the age of 60, individuals with hypertension, individuals without diabetes mellitus, individuals without fatty liver disease, and individuals who were smokers (all *P* values < 0.05). However, the *P*-value for interaction > 0.05 indicates that there is no significant interaction across groups.

### Restricted cubic spline curve fitting

**Fig. 3:**
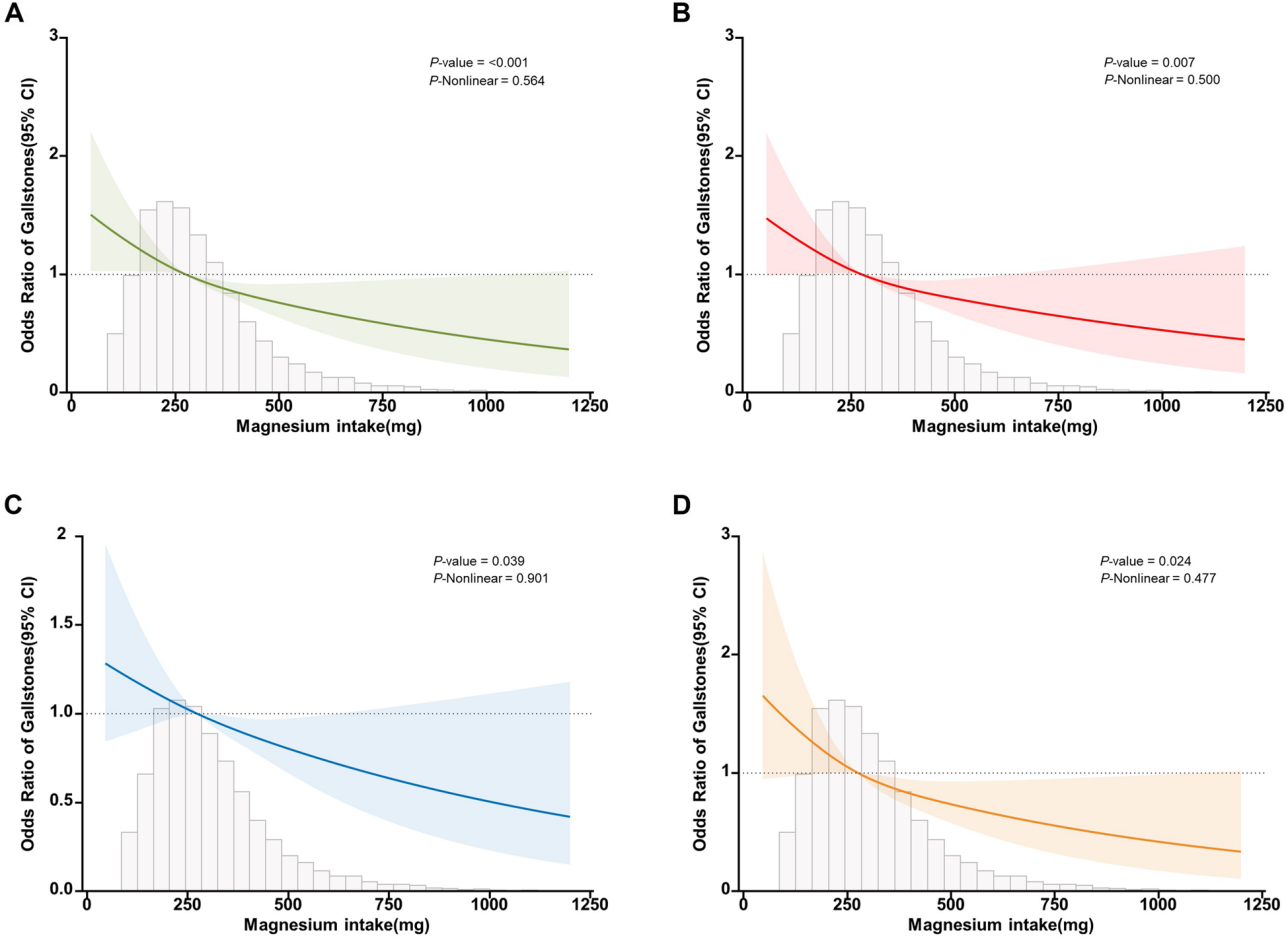
RCS curve fit between magnesium intake and gallstones. Notes: Solid lines represent smooth curve fits between variables. Shaded bands represent 95 per cent confidence intervals from the fit. Subfigure A: no adjustment for covariates. Subfigure B: adjusted for sex, age, and race. Subfigure C: adjusted for sex, age, race, education level, marital status, PIR, BMI, hypertension, diabetes mellitus, fatty liver, smoking, alcohol consumption, TG, HDL-C, TC, ALT, AST, and GGT. Subfigure D: adjusted for sex, age, race, education level, marital status, PIR, BMI, hypertension, diabetes mellitus, fatty liver, smoking, alcohol consumption, TG, HDL-C, TC, ALT, AST, GGT, protein intake, carbohydrate intake, fat intake, water intake, calcium intake, iron intake, and zinc intake

Figure 3 depicts the relationship between magnesium intake and gallstones using a restricted cubic spline curve (RCS), and the density of distribution of participants according to magnesium intake. Subfigures A to D depicts the dose-response relationship between magnesium intake and gallstones after adjusting for different covariates, respectively. As magnesium intake increased, the incidence of gallstones gradually decreased. Overall, there was a linear negative correlation between magnesium intake and gallstones, which was particularly evident in subfigure C (Nonlinear *P* = 0.901).

### The intermediary role of AIP

**Fig. 4:**
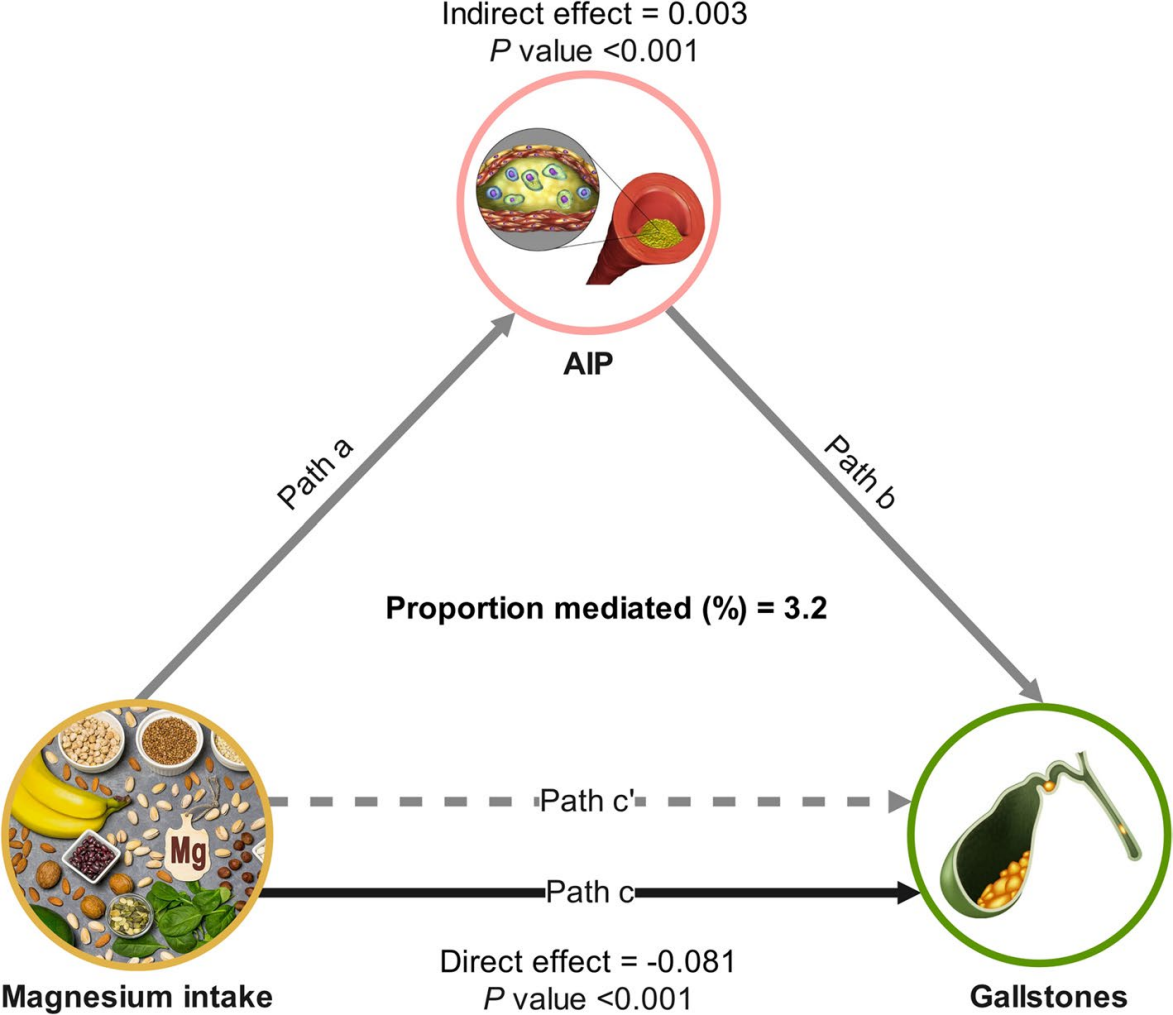
Mediated analysis model path diagram. Notes: Dietary magnesium intake was defined as the independent variable; gallstones as the dependent variable; and AIP as the mediating variable. Path a represents the regression coefficient of the association between dietary magnesium intake and AIP. Path b represents the regression coefficient of the association between AIP and gallstones. Path c represents the simple total effect of dietary magnesium intake on gallstones. Path c' represents the direct effect of dietary magnesium intake on gallstones when controlling for AIP

Causal mediation analysis was conducted to explore the potential mediating role of AIP in the relationship between dietary magnesium intake and the development of gallstones. Figure 4 depicts the mediation model and pathway, where magnesium intake is treated as the independent variable, AIP as the mediator, and gallstones as the dependent variable. The findings indicated that dietary magnesium had a notable indirect impact on the prevalence of gallstones via AIP, exhibiting an indirect effect size of 0.003 (95% CI: 0.001, 0.005; *P* < 0.001), indicating that AIP served as a partial mediator in the association between magnesium and gallstones. Furthermore, even after adjusting for AIP, the relationship between dietary magnesium intake and gallstones remained statistically significant (*P* < 0.001), demonstrating a significant direct effect. This suggests that there are both direct and indirect effects of AIP on dietary magnesium intake and gallstone occurrence, with approximately 3.2% being mediated by AIP. The results of the mediation analyses, including direct effects, indirect effects, total effects, and mediation ratios, are presented in Table 5.

**Table 5:** Mediation analysis of AIP in the association between magnesium intake and gallstones

Independent variable	Mediator	Total effect		Indirect effect		Direct effect		Proportion mediated, %
		Coefficient (95% CI)	*P* value	Coefficient (95% CI)	*P* value	Coefficient (95% CI)	*P* value	
Magnesium intake	AIP	-0.079 (-0.122, -0.034)	<0.001	0.003 (0.001, 0.005)	<0.001	-0.081 (-0.125, -0.036)	<0.001	3.2%

## Discussion

The present study provides new insights and findings on the mediating role of AIP in the association between dietary magnesium intake and gallstones. By examining a sample of 4,841 adults, a strong association was found between the consumption of magnesium-rich foods and a lower incidence of gallstones, which could potentially be attributed to the role of magnesium in improving blood lipid levels. It is worth noting that this negative correlation association remains stable after gradually adjusting for confounding covariates from Model 1 to Model 4. Subgroup analyses showed that the effect of magnesium intake on gallstones was more pronounced in women, hypertensive patients, non-diabetic patients, patients without fatty liver disease, and smokers. A linear negative correlation was found between dietary magnesium intake and the occurrence of gallstones by using RCS curve fitting. Overall, adequate magnesium intake reduces the incidence of gallstones to some extent, and this negative correlation is partly mediated by AIP.

In recent years, more and more scholars have begun to focus on the biological and lipid markers associated with gallstones, and have gradually reported the potential associations between gallstones and a variety of factors such as metabolism and environment. Li et al. [32] conducted a cross-sectional study that revealed a positive correlation between elevated cobalt blood levels and increased prevalence of gallstones, particularly among female individuals, those aged 60 and above, individuals with hypertension, and individuals with diabetes. Ke and colleagues' study [33] revealed a significant correlation between weight-adjusted waist circumference index (WWI) and heightened susceptibility to gallstones among adults in the United States. Upon thorough adjustment for potential confounding variables, it was determined that a one-unit rise in WWI corresponded to a 34% rise in the likelihood of developing gallstones. These findings indicate that WWI may offer greater predictive utility in clinical settings compared to traditional measures of obesity and metabolic health. Chen et al. [34] conducted a fundamental study demonstrating the critical role of proprotein convertase subtilisin/kexin type 9 (PCSK 9) in the pathogenesis of gallstones. PCSK 9, a serine protease, plays a crucial role in regulating cholesterol metabolism and its dysregulation has been associated with the development and progression of gallstones. As the levels of PCSK9 increase in the liver of individuals with gallstones, there is a corresponding increase in cholesterol levels, exhibiting a negative correlation with bile acid levels[35, 36]. Inhibitors of PCSK 9 offer a promising therapeutic strategy for promoting cholesterol conversion to bile acids, thereby presenting a novel target for the clinical management of gallstones. Moreover, a recent systematic review indicates that PCSK9, beyond its role in lipid metabolism, is intricately linked to various diseases including atherosclerosis, central nervous system disorders., sepsis, and chronic renal failure [37]. The above findings highlight the rationale for incorporating multiple indicators when assessing gallstone risk and provide a basis for elucidating the intricate etiology of gallstones. However, the current scholarly literature lacks a thorough exploration of the potential link between dietary magnesium and gallstones, and it remains uncertain whether magnesium mitigates gallstone risk through modulation of AIP (a new biomarker for lipid metabolism disorders and inflammation).

This study is an original use of a public database to examine the relationship between gallstones and dietary magnesium intake and to explore the potential moderating role of AIP. In previous epidemiological studies, a variety of systemic diseases have been studied in depth about AIP. In a large prospective cohort study, Zhang et al. [38] observed a 30% rise in the likelihood of myocardial infarction with each incremental unit increase in AIP. This indicates that prolonged elevation of AIP levels may heighten the likelihood of myocardial infarction within the general populace. Through a cross-sectional examination of 7,017 Chinese subjects, Xu et al. [39] identified a significant correlation between AIP and hyperuricemia. Even after excluding individuals taking long-term lipid-lowering medications, the findings remained consistent in sensitivity analyses. AIP exhibited superior discriminatory capability compared to other lipid markers in the prediction of hyperuricemia. Lin et al. [40] demonstrated that AIP served as a robust independent risk factor for NAFLD among patients with type 2 diabetes. In addition, they assessed the diagnostic ability of AIP for NAFLD using a subject operating characteristic curve with a specificity and sensitivity of 90.1% and 65%, respectively. In addition to being associated with atherosclerosis, AIP, as a novel lipid marker, has also been associated with a variety of systemic diseases.

There is evidence of a link between dietary magnesium intake and lipid metabolism. In both animal and clinical investigations, diets lacking in magnesium have been found to increase levels of triglycerides in the bloodstream while simultaneously decreasing levels of HDL-C [41–45]. HDL-C is a factor that provides protection against cardiovascular disease and is significantly and inversely associated with the extent of arterial lumen stenosis [46, 47]. HDL-C facilitates the transportation of cholesterol to surrounding tissues, where it is converted to bile acids or directly from the bile through the intestines to the biliary system, thereby reducing the risk of gallstones [48]. Jin and colleagues performed a retrospective analysis on a cohort of 12,284 participants surveyed between 2001 and 2013, utilizing data from the NHANES database. The findings indicated a positive correlation between magnesium intake and HDL-C levels among female individuals. In contrast, magnesium intake was negatively related to TG levels in both men and women [49]. AIP is a quantitative metric utilized for evaluating lipid metabolism through the integration of triglycerides and HDL cholesterol. This index provides valuable information about the ratio of triglycerides to HDL cholesterol and the size of lipoprotein particles. Nonetheless, the precise mechanism through which dietary magnesium intake influences AIP levels and the mediating role of AIP in heightening susceptibility to gallstones remain uncertain and can be elucidated through various potential biological mechanisms. Firstly, magnesium regulates the activation of genes associated with lipid degradation, such as peroxisome proliferator-activated receptors (PPARs), and a deficiency in dietary magnesium promotes the generation of oxygen-free radicals, resulting in an elevation in the secretion of mucus glycoproteins in the gallbladder [45, 50, 51]. Secondly, magnesium regulates the activity of lipoprotein lipase (LPL), a key enzyme involved in the hydrolysis of triglycerides from circulating lipoproteins, thereby regulating blood triglyceride levels. Studies have shown that magnesium improves lipoprotein clearance, thereby increasing the clearance of triglyceride-rich lipoproteins and lowering plasma triglycerides, which further affects AIP levels [52]. According to Andreotti et al. [53], there was a significant correlation between elevated TG levels, decreased HDL-C levels, and an elevated susceptibility to gallstones. Finally, magnesium plays a significant role as a primary coenzyme in numerous enzymatic processes and serves as a crucial facilitator of insulin's impact on cells [53, 54]. Insufficient magnesium disrupts the enzymatic processes responsible for generating adenosine triphosphate and the required tyrosine kinase activity for insulin function. Additionally, it modifies the enzymatic reactions related to glucose metabolism, resulting in the development of insulin resistance [55–57]. Yin et al. illustrated a noteworthy, non-linear, positive correlation between insulin resistance and AIP. Reduced insulin sensitivity and elevated concentrations are the main features of insulin resistance, which may increase AIP levels as well as cholesterol saturation in bile, thereby promoting gallstone formation [58, 59].

The current study offers compelling evidence supporting a relationship between dietary magnesium intake and gallstones, as well as establishing a connection with the lipid metabolism marker AIP. Given that TG and HDL-C can be derived from standard blood biochemical markers, the calculation of AIP is both cost-effective and easily implemented in clinical settings. Furthermore, the direct calculation of AIP through a formula circumvents the need for intricate physical examinations and laboratory procedures, thereby enhancing its utility in the early detection of gallstone risk. This suggests to clinical providers that promoting a sensible diet should be a priority for patients with persistently high AIP. Appropriate supplementation with magnesium-rich foods or magnesium supplements is a proven method of preventing gallstones.

## Strengths and limitations of the study

This study demonstrates multiple strengths. Firstly, it is the inaugural cross-sectional study to examine how magnesium intake correlates with gallstone risk, incorporating lipid parameters for causal mediation analyses, thus offering novel perspectives on gallstone prevention and treatment. Secondly, the data utilized in this study came from a comprehensive database containing a large population sample, thus enhancing the representativeness of the findings. Lastly, the statistical adjustment of covariates in the model was conducted rigorously, resulting in more robust and persuasive conclusions. This study has some limitations, of course. Firstly, the diagnosis of gallstones was based only on responses to a medical health questionnaire and lacked a more precise imaging diagnosis. Secondly, dietary data was obtained through a 24-hour food interview, which may not accurately reflect long-term dietary habits and could be influenced by recall bias. Third, serum magnesium information was not collected from participants in the NHANES survey conducted between 2017 and March 2020, and whether dietary magnesium intake may in turn affect gallstone formation by altering serum magnesium levels needs to be explored in future more comprehensive prospective cohort studies.

## Conclusion

According to this study, dietary magnesium intake had a significant linear negative association with the prevalence of gallstones, in which AIP played a mediating role. This finding now provides valuable recommendations for the prevention and treatment of gallstones, enlightening healthcare professionals that rational supplementation of magnesium-containing foods may reduce the risk of gallstones risk by modulating lipid metabolism.

## Data Availability

No datasets were generated or analysed during the current study.

## Abbreviations

NHANES: National Health and Nutrition Examination Survey
BMI: Body mass index
NCHS: The National Center for Health Statistics
CDC: Centers for Disease Control and Prevention
CI: Confidence interval
SD: Standard deviation
CAP: Controlled attenuation parameters
VIF: Variance inflation factor
IQR: Median and interquartile range
OR: Odds ratio
PIR: Ratio of household income to poverty
AIP: Atherogenic index of plasma
ALT: Alanine aminotransferase
GED: General equivalency diploma
TG: Triglyceride
AST: Aspartate aminotransferase
GGT: Gamma glutamyl transferase
TC: Total cholesterol
HDL-C: High-density lipoprotein cholesterol
PCSK 9: proprotein convertase subtilisin/kexin type 9
